# Reliable Top-Left Light Convention Starts With Early Renaissance: An Extensive Approach Comprising 10k Artworks

**DOI:** 10.3389/fpsyg.2018.00454

**Published:** 2018-04-09

**Authors:** Claus-Christian Carbon, Alexander Pastukhov

**Affiliations:** ^1^Department of General Psychology and Methodology, University of Bamberg, Bamberg, Germany; ^2^Forschungsgruppe Ergonomics, Psychological Æsthetics, Gestalt, Bamberg, Germany

**Keywords:** empirical aesthetics, light source, renaissance, artworks, art history, laterality, art and science

## Abstract

Art history claims that Western art shows light from the top left, which has been repeatedly shown with narrow image sets and simplistic research methods. Here we employed a set of 10,000 pictures for which participants estimated the direction of light plus their confidence of estimation. From 1420 A.D., the onset of Early Renaissance, until 1900 A.D., we revealed a clear preference for painting light from the top left—within the same period, we observed the highest confidence in such estimations of the light source.

**One sentence summary:**

This study demonstrates a robust preference for painting light from the top left for Western art history, starting from Early Renaissance until 1900.

## Introduction

**Figure 1:**
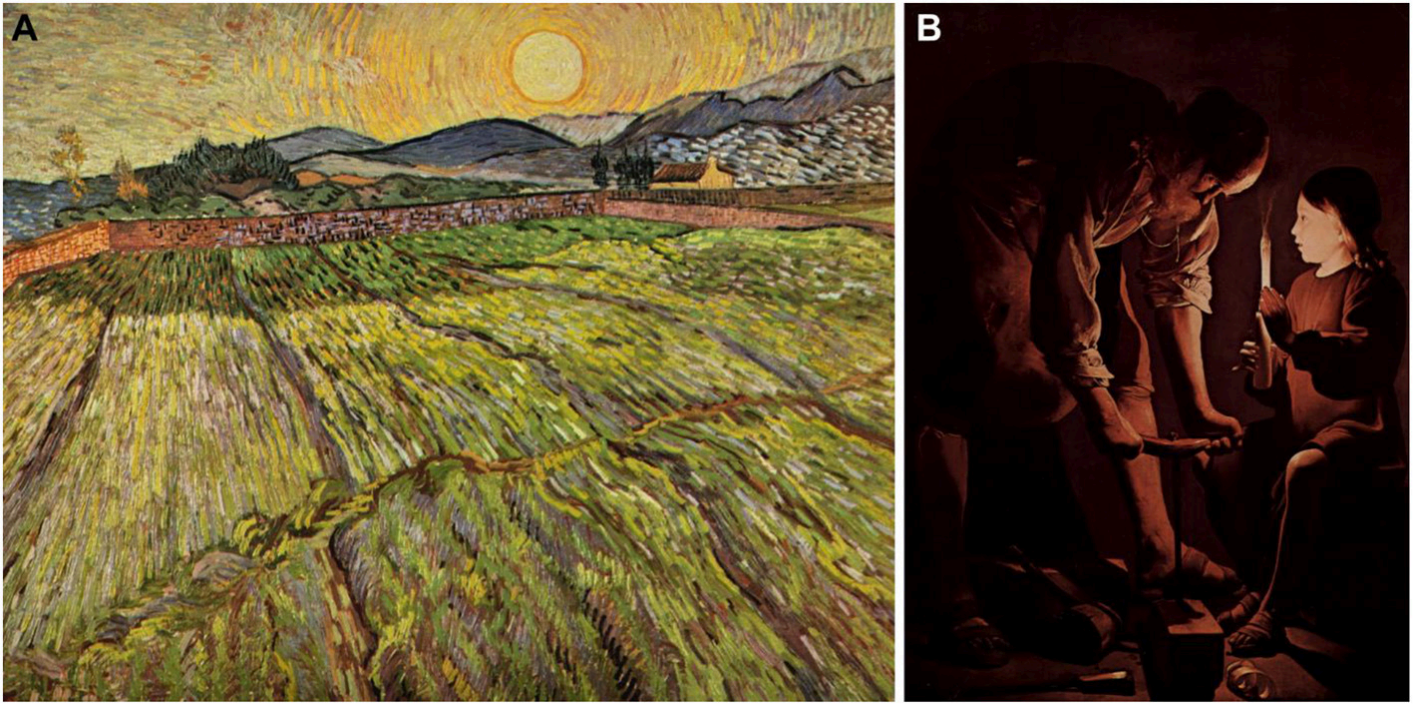
**(A)** Painting “Enclosed field with rising sun” by Vincent van Gogh from the year 1889, explicitly showing the source of light—the sun. **(B)** Painting “Joseph the Carpenter” by Georges de La Tour from the approximate year 1645 explicitly showing the source of light—a candle. Both pictures and their reproductions are in the public domain (Creative Commons CC-BY license).

“Light creates space”—this is how art theorist and perceptual psychologist Rudolf Arnheim boiled down the essential meaning of depicting light in paintings (Arnheim, 1974). However, space comes along with the possibility of disambiguating the shape of objects, so light also assists the perception of three-dimensional structures. This disambiguation is not very effective as long as the location of the light source is unknown or unreliably assessed (Rock, 1983). There are only rare cases where we can directly observe the source of light in paintings, e.g., explicitly showing the sun as is often done in Van Gogh's Wheat Field series of oil paintings (see Figure 1A), or by showing a human-made light source such as candles in the famous Georges de La Tour paintings (see Figure 1B). In most other cases we have to infer the light source from the characteristic brightness gradient/shading or, alternatively, rely on the conventions realized in certain art historical contexts (Gerardin et al., 2010). Cavanagh and Leclerc (1989) showed that the assumption of light coming from above assists the consistent interpretation of shape aspects in a visual scene.

Art historian Gombrich, who was very much devoted to perceptual psychology, reported that (Western) artists preferred light from the (top) left. He identified the simple fact that artists were mostly right-handers as a major reason for this bias (Lanthony, 1995). This way the drawing hand does not block the emitted light (Gombrich, 2002). Gestalt psychologist Metzger's *Gesetze des Sehens* might be the major source for these claims (Metzger, 1953). The connection between handedness and the preference for lighting direction was empirically shown by Sun and Perona (1998) but was challenged by later studies (Mamassian and Goutcher, 2001).

Very early attempts to test for a (top-) left bias of light sources focused on Western paintings, specifically on Western portraits analyzing specific preferences for the left or right profile (Humphrey and McManus, 1973; McManus and Humphrey, 1973). Coles (1974) demonstrated a clear bias for the direction of lighting from a left to right direction in portraits. A recent study on frequencies of leftward vs. rightward depictions in Korean newspapers added to the impression (Lee and Oh, 2015). Korean culture had undergone a direction change of reading and writing over the last century, from leftward to rightward. The research revealed an ongoing, concordant change of depictions of drawings, but not of photos, during this period. McManus addressed laterality effects on artworks (McManus, 1979, 2005; McManus et al., 2004). By taking 175 cases of Medieval and Renaissance crucifixions into account, he observed a clear decline in the number of pictures with the light straight on or where the light source is depicted rather ambiguously. Further analyses of the direction of light in Renaissance Madonna-and-Child paintings demonstrated a significant increase in pictures with light from the right, but nevertheless from 1250 until 1549 A.D. the most frequent direction was from the left (with a *number* of pictures per bin spanning 50 years, ranging from 9 to 394). Subsequent work by Mamassian (2008) replicated the general finding of a predominance of left-lit paintings when observing portraits (*k* = 194) as well as non-portraits (*k* = 465), selectively employing artworks from the Louvre museum in Paris.

The present study aimed at widening up the view on the documented left bias of Western artworks by expanding the already existing studies. First, McManus's seminal studies focused on very specific artistic *genres*; actually crucifixions and Madonna-and-Child paintings, which led to the question of how the findings are transferable to other artistic topics. Secondly, although McManus took great effort to include many paintings in his studies, the number per time range bin was still clearly limited: in some cases *k* < 10–20 paintings were employed to cover a range of 50 up to 100 (and even 550) years. Third, the origin of the paintings was quite narrow due to the *sujets* utilized, focusing only on Italy (i.e., Florentine, Venetian, Central and Northern Italian art). Last but not least, the method of analyzing whether a painting shows a left vs. right (vs. neutral) direction of light source shows and yields clear limitations: (a) It is used in a dichotomous (left vs. right, Coles, 1974) or tripartite (left vs. right vs. neutral, McManus, 1979) way without further differentiation, (b) descriptions of exactly how the assessment of the direction of the light source was conducted is missing, also bringing into question whether the assessment was checked via observer consensus statistics, and (c) a control condition is missing for checking the base rate of lateral assessment [it could be possible that assessments are already biased due to the knowledge from courses on Western art, where the standard of a top-left light source is typically taught and taken for granted (Mamassian and Goutcher, 2001; Gombrich, 2005; Stone et al., 2009)].

Therefore, we employed an empirical study where different participants had to assess the direction of the primary light source, painting by painting. We utilized more than 10,000 high-quality depictions of paintings from a broad span of time, representing a major part of Western art history with a richness of themes from all areas of Europe; so not just from specific regions, painters, epochs, art genres or art galleries. Importantly, we developed a more sophisticated way of assessing the direction of light source. First, we employed art-naïve persons who had to assess the source of light by drawing the supposed direction of the light beam to gain more fine-graded data. This paradigm, where different persons had to assess the same pictures, also allowed us to test for inter-rater reliability. Second, previous results could have been biased when assessing the laterality of the light source due to mental models of where the light should typically be coming from (actually, as often taught in school, “from the top left”). In addition, over time, participants might develop a response bias when being confronted over-representatively frequently with a certain light condition in pictures. As a counter-action, we decided to mirror half of the presented stimuli horizontally to expose the participants to a mixture of original and reflected images. This enabled us to test for pre-set and response biases toward the assessment of the light source and to assess the reliability of assessments. Third, some paintings are showing more or less valid cues of light sources. We were interested in analyzing the factor of unambiguousness by employing an additional measure, which asks for the confidence in the light-source assessment. The main idea behind this was that at a certain period in art history, not only did a specific direction of light source became standard, but also the means to show the source became more sophisticated and so more unambiguous over time.

## Materials and methods

### Paintings set

Paintings were all taken from the Wikimedia Commons Yorck Project (https://commons.wikimedia.org/wiki/Category:PD-Art_(Yorck_Project)) and are available under the *Creative Commons Attribution-ShareAlike License*. The initial paintings list consisted of 10,365 entries, but we excluded any paintings with incomplete meta-information or ones which we failed to acquire via an automated script. The final list consisted of 9,533 paintings of which 9,469 were presented to observers (paintings were chosen randomly at runtime, hence we faced the situation that some paintings were never selected for estimation, actually *k* = 64). Artworks were from a broad time interval, spanning from ~1500 B.C. to 2000 A.D., although most were created between 1300 A.D. and 1950 A.D. See also Table 1 for the distribution of the paintings' geographical origin.

**Table 1:** Number of paintings from various locations.

**Country of origin**	**Number of paintings**
Unknown	3,120
Italy	1,990
France	1,738
Germany	1,050
The Netherlands	935
Great Britain	375
Spain	320
Austria	227
Russia	89
China	85
Switzerland	79
USA	66
Romania	59
India	58
Egypt	29
Bohemia	25
Turkey	17
Byzantium	16
Greece	12
Denmark	11
Japan	10
Sweden	8
Ireland	7
Macedonia	5
Norway	5
Orient	5
Poland	4
Belgium	4
Serbia	3
Asia Minor	2
Persian	2
Hungary	2
Syria	2
Africa	2
Portugal	1
Finland	1
Bulgaria	1

### Observers

Seven participants (four female; *M*_age_ = 22.7 years, all right-handed, see Table 2) participated in the measurement. All participants had a normal or corrected-to-normal vision (assessed by a standard Snellen eye chart test) and normal color vision (assessed by a short version of the Ishihara color test). Participants were students from the University of Bamberg and received partial course credit for their participation. They had no prior experience with the present task and were naïve to the purpose of this experiment; they did not obtain specific training in art history. All procedures were in accordance with the Declaration of Helsinki. The study was in full accordance with the ethical guidelines of the University of Bamberg and was approved by the university ethics committee on 18 August 2017.

**Table 2:** Summary for the individual observers.

**Observer**	**Sex**	**Age**	**No. of trials**	**Proportion of trials with an estimate (%)**	**Proportion of mirror-corrected estimates to originate on the left**			**Relative orientation, degrees**	**Confidence**
					**Lower 99% *CI***	***M***	**Upper 99% *CI***	***M* ± *SD***	***M* ± *SD***
HHA96w	f	20	4,882	71.3	63.7	65.8	67.9	55.3°± 23.0°	4.8 ± 0.9
IKB95w	f	21	1,270	76.2	64.4	68.3	72.1	55.6°± 19.8°	5.4 ± 1.3
KSC94w	f	22	63	76.2	38.9	58.3	76.1	71.0°± 29.5°	4.1 ± 1.3
MMN92m	m	24	202	62.4	50.9	62.7	73.5	93.0°± 39.4°	4.5 ± 1.0
SEF89m	m	27	248	85.1	52.1	61.1	69.7	72.4°± 41.8°	4.0 ± 1.2
SKL94w	f	22	9,358	87.6	66.3	67.6	68.9	39.1°± 12.6°	4.7 ± 1.6
SSK93m	m	23	486	78.0	58.6	65.2	71.4	38.8°± 23.3°	4.8 ± 1.0

*99% confidence interval (CI) for the proportion of mirror-corrected estimates to originate on the left is a 99% binomial CI. Relative orientation shows the angle relative to the vertical irrespective of the light source origin*.

### Apparatus

Participants estimated a direction of light in paintings using a custom program written in Python. Due to the extremely large number of evaluations required from each participant, they were allowed to run it on their personal laptops. Settings were adjusted for each individual computer to ensure comparable display size across all devices.

### Procedure

**Figure 2:**
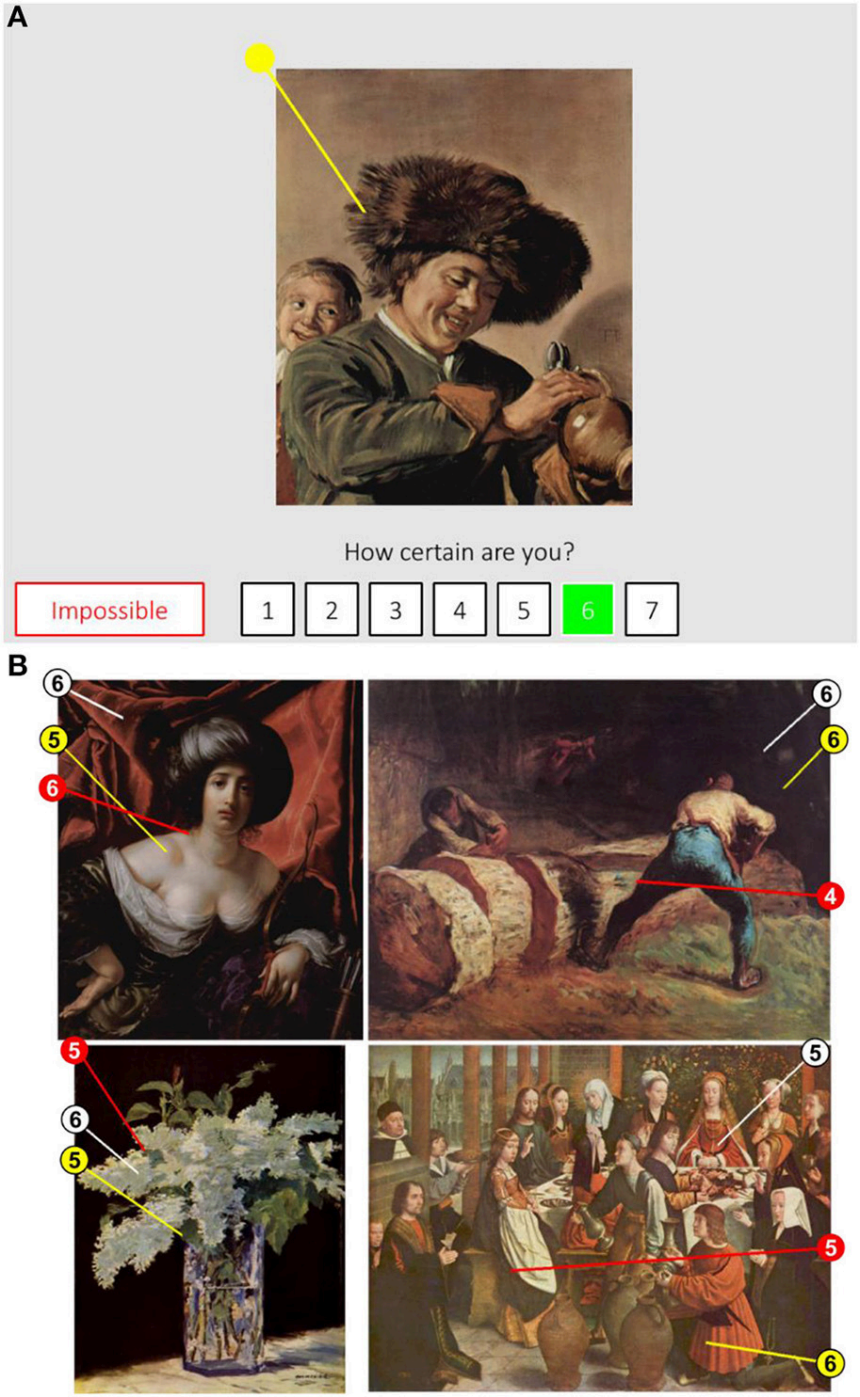
**(A)** A screenshot of the experimental program. See text for details. **(B)** Examples estimates of the approximate direction of light in the paintings. Numbers depict participants' confidence.

A screenshot of the program is depicted in Figure 2A. Participants used a mouse to draw an estimate of the approximate origin and direction of light in the painting (yellow vector in Figure 2B, circle depicts the origin). Paintings were presented either in their original orientation or flipped horizontally to minimize a potential build-up of the response bias (random selection with 50% of correct and flipped orientation). Accordingly, in the results section we present both raw, uncorrected estimates (as submitted by participants) and mirror-corrected estimates. Viewing time was unlimited and observers were instructed to prioritize accuracy of response over speed. To complete the task, participants indicated their confidence about the estimate using a 1 to 7 rating scale (1 = *very uncertain*, 7 = *very certain*). Participants had the opportunity to forgo an estimate if they felt that for that specific painting estimating light source was impossible.

### Statistical analysis

Data was preprocessed using custom software written in Python. The statistical analysis was carried out using R software (R Core Team, 2017). Linear-mixed modeling was performed using lmerTest package (Bates et al., 2015; Kuznetsova et al., 2016). Binomial confidence intervals were computed via binom package (Dorai-Raj, 2014).

### Data availability

All data files and the code that was used to conduct statistical analyses and produce figures for the paper are freely available at https://osf.io/t5qfp.

## Results and discussion

**Figure 3:**
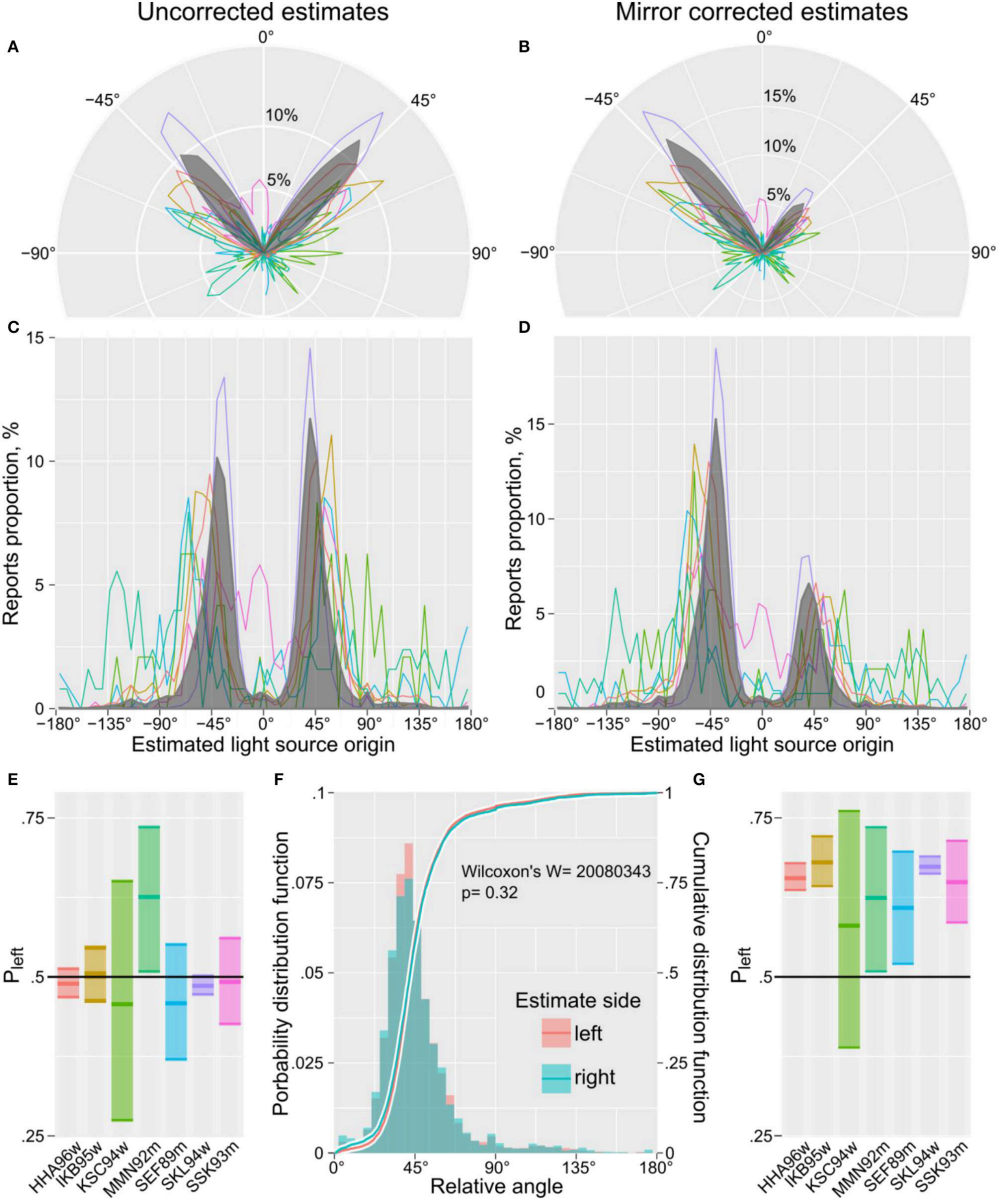
The estimated angles of illumination for **(A,C)** the raw uncorrected estimates and **(B,D)** the mirror corrected estimates. Plotting conventions: 0° corresponds to the light source being positioned directly above the paintings. Solid lines depict individual observers, gray area depicts the overall group responses. **(E,G)** Proportion of estimates left off the vertical (*P*_left_, mean and 99% binomial confidence interval). **(F)** Distribution of the relative angle for light source estimates with light originating on the left and right side.

First, we tested for potential biases in assessing the light source with a simple check-up of the data: As we randomly (horizontally) mirrored 50% of the presented pictures we were able to check the overall distribution of assessments, which was nearly perfectly balanced around 0° (see Figures 3A,C,E and Table 2). Importantly, once the reports were corrected for the mirroring, there was a clear and strong preference for observers to report light the source as being located on the left (see Figures 3B,D,G and Table 2). However, the orientation of estimates relative to the vertical did not depend on whether the estimate was left- or right-sided (Figure 3F and Table 2). This pattern of results validates our procedure and confirms the prior reports of the light in paintings mainly originating from the left.

**Figure 4:**
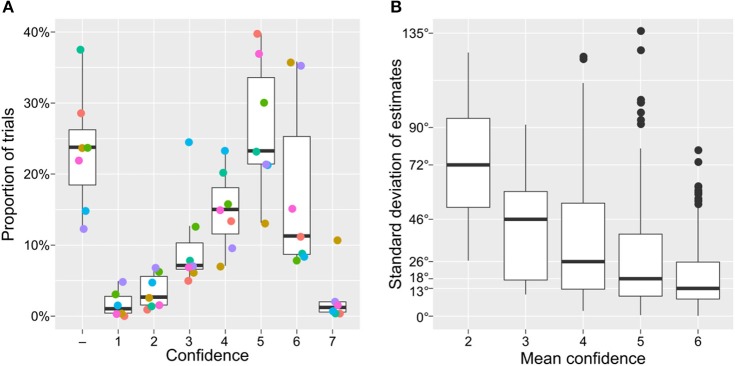
Distribution of confidence reports and observer consistency. **(A)** The proportion of confidence reports, circle colors denote individual observers. **(B)** The standard deviation of estimates as a function of average reported confidence level (only paintings with estimates from at least three different participants were included in the analysis). Higher confidence resulted in estimates that were more consistent across observers.

With respect to the observers' confidence, we found that they tended to report an estimate only when being confident about it (see Figure 4A, ~80% of trials with an estimate had a confidence rating of four or above, from 1 = *min* to 7 = *max*). Higher confidence was associated with significantly more consistent estimates across participants, see Figure 4B. To quantify the effect, we restricted the analysis only to paintings with an estimate from at least three different participants. Specifically, we fitted a linear model with a standard deviation of estimates for individual paintings as a dependent variable and an average observers' confidence as a fixed factor. The analysis showed a strong negative relationship, *t*_(745)_ = −9.8, *p* < 0.0001, *R*^2^ = 0.34, confirming the idea that paintings with richer light source information led to both higher confidence and more consistent estimates.

**Figure 5:**
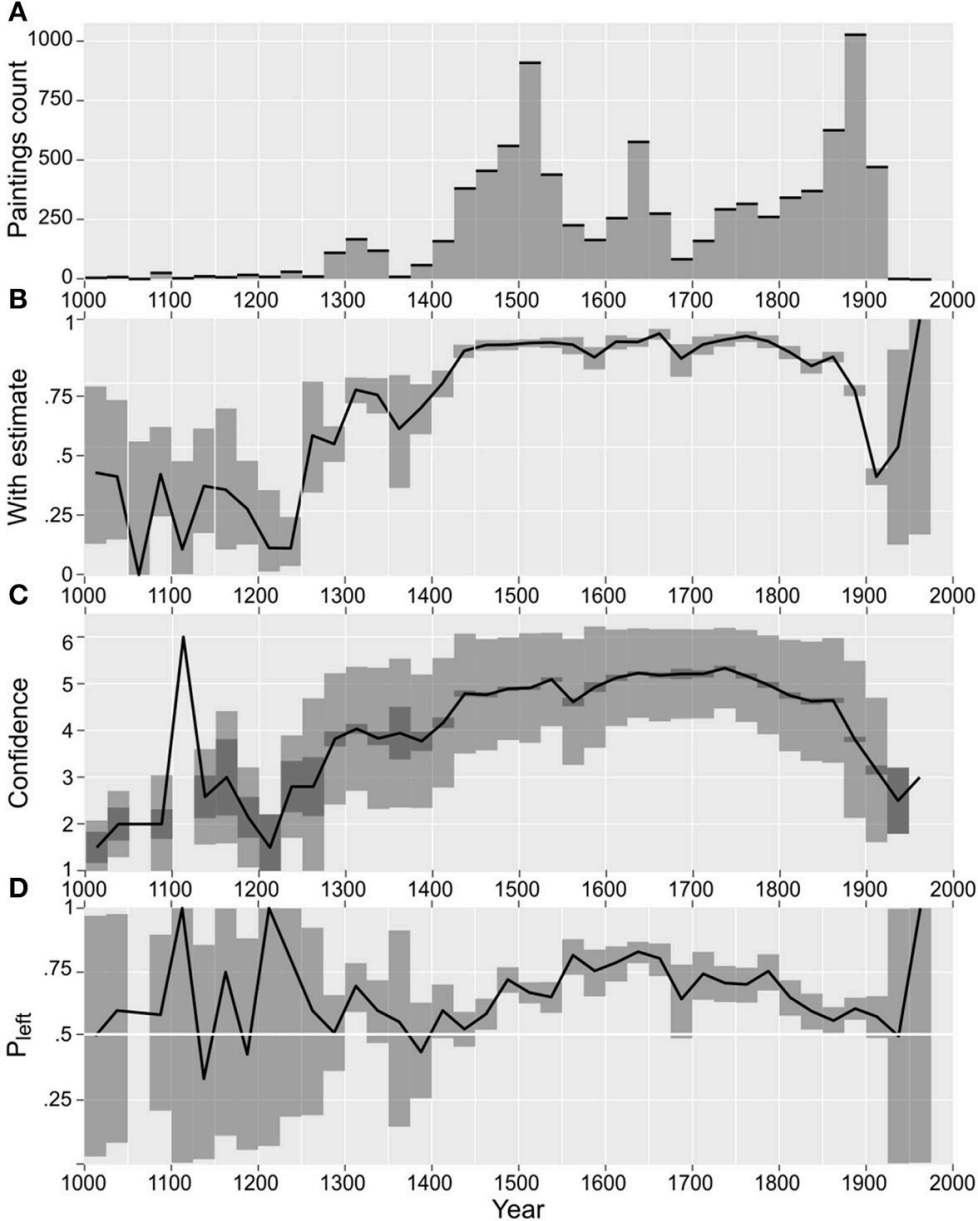
Light source Zeitgeist. **(A)** Distribution of paintings' creation date. Please note that paintings created before 1000 A.D. are not shown. **(B)** The proportion of paintings for which an estimate was possible. **(C)** Participants' confidence, lighter and darker gray stripe denote, respectively, standard deviation and standard error of the mean. **(D)** The proportion of the light source direction estimated as originating from the left. Gray stripe denotes 99% binomial confidence interval.

We also analyzed for Zeitgeist-dependent positioning of the light source in paintings (we used the approximate dating of paintings with a resolution of 25 years; see data and statistics Figure 5). We found that the preferential location of the light source was synchronized with the beginning of the Early Renaissance era, starting at ~1420 A.D. and on through the *Cinquecento* until the end of the nineteenth century. Our data shows that from the Renaissance on, Western art history had “its bias” to the left regarding the light source. Paintings for which we found clear initial, but also singular, laterality effects were created by Simone Martini (1284–1344), Giotto (1266/1267–1337), and Duccio di Buoninsegna (1255/1260–1318/1319). This is very compatible with the notion of art historians that lighting and shadowing effects were identified as a basic and innovative topic of Western art history with the rise of Early Renaissance painters such as Masaccio (1401–1428), Andrea Mantegna (1431–1506), and Andrea del Verrocchio (1435–1488). Important techniques to realize lighting and shadowing effects were developed during this period; most importantly was a technique where strong contrasts between light and dark are used-known as *chiaroscuro* (from Italian *chiaro*, “light,” and *scuro*, “dark”). Critically, some preliminary works also dealing with clear lighting and shadowing effects could have been covered in the statistical analysis by the mere fact that before 1400 A.D., a comparatively smaller number of paintings were (and are) available (see Figure 5A). Still, based on participants' confidence (Figures 5B,C), we can clearly state that overall there was no clear and reliable way of positioning the light source before the Early Renaissance.

## Conclusions

By employing an extensive set of images of very different *sujets* of Western art history, we have compiled clear and unbiased evidence that within the period 1420–1900 A.D., painters preferred to paint the light source from the top left. This result, based on participants' estimates, was complemented by the participants' confidence in their estimates. Fin de siècle ended this 500-year-long dominance in art history, opening new avenues of artistic depictions of light, contrast, and depth.

## Author contributions

CCC had the initial idea; CCC and AP contributed conception and design of the study; AP programmed the procedure; AP performed the statistical analysis; CCC and AP wrote the first draft of the manuscript. All authors contributed to manuscript revision, read, and approved the submitted version.

### Conflict of interest statement

The authors declare that the research was conducted in the absence of any commercial or financial relationships that could be construed as a potential conflict of interest.
